# Alcohol use disorder and sleep disturbances: Current perspectives

**DOI:** 10.1192/j.eurpsy.2023.353

**Published:** 2023-07-19

**Authors:** C. Afonso Romano, S. Neves Martins, J. Amaral

**Affiliations:** Psychiatry and Mental Health Department, Tâmega e Sousa Hospital Centre, Oporto, Portugal

## Abstract

**Introduction:**

Alcohol Use Disorder (AUD) is one of the most significant public health problems in Europe, being highly associated with several medical and psychiatric comorbidities. Sleep disturbances are in this interface and may include insomnia, alterations of sleep architecture and circadian rhythm abnormalities, breathing-related sleep disorders, and sleep-related movement disorders. Also, considering the three stages of the addiction cycle (binge/intoxication, withdrawal/negative affect, and preoccupation/anticipation) and since these domains are reflected in key regions of the brain, it is possible to map these nearly ubiquitous sleep disturbances.

**Objectives:**

This review aims to summarize the current literature related to the association between sleep disorders and AUD, with a focus on its clinical aspects and neurobiology.

**Methods:**

Non-systematic research was made recurring to the PubMed database, with the keywords “alcohol use disorder”, “sleep”, “sleep disorder”. The most relevant articles were selected, focusing on articles published in the last decade.

**Results:**

In patients with AUD, the prevalence of insomnia ranges from 36-91%. A possible mechanism underlies in a mismatch involving maintained activity in wake-promoting structures during non-rapid eye movement sleep (NREM) and a blunted homeostatic drive. On the other hand, alcohol consumption also affects the normal sleep-wake cycle, due to a disruption in the underlying circadian rhythms, a mechanism compassed by the suprachiasmatic nucleus and by photic and non-photic cues. Considering this, it seems highly likely that insomnia and circadian abnormalities may coexist in some individuals. Moreover, AUD is implicated in initiation or worsening of breathing-related events in sleep, especially when having a history of snoring or sleep apnoea syndrome and in period limb movement disorder.

Simultaneously, sleep disorders in AUD can be incorporated into the three-stage addiction cycle. In the binge/intoxication stage, excessive alcohol intake leads to a faster sleep onset but poor sleep quality, explained by the effects on GABAergic systems. During the withdrawal/negative affect stage, there is a decrease in slow-wave sleep and limited rapid eye movement (REM) sleep recovery, which can be explained by the alcohol-positive allosteric modulation of GABA_A_ receptor and other mechanisms. Lastly, during the preoccupation/anticipation stage, the glutamatergic system dysregulation contributes to persistent sleep disturbances, including insomnia, decrease in slow-wave sleep, and an increase in REM sleep.

**Conclusions:**

The knowledge of sleep disturbances associated with AUD has grown and has suggested a bidirectional process that appears to play an essential role in the addiction process. Further studies investigating this association are warranted.

**Disclosure of Interest:**

None Declared

